# Confirmation of Attraction of the Mixture of a Sugar–Vinegar–Alcohol Lure and Methyl Eugenol for Sexually Immature Males of *Bactrocera dorsalis*

**DOI:** 10.3390/insects17070707

**Published:** 2026-07-08

**Authors:** Dian Zhou, Fang Fang, Shaozhi Wang, Huating Lu, Qiong Kong, Shengyong Yuan, Chun Xiao

**Affiliations:** 1 Faculty of Plant Protection, Yunnan Agricultural University, Kunming 650201, China; zhoudian_hn@163.com (D.Z.); fangf3264@163.com (F.F.); dxdwsz@163.com (S.W.); lht202503@163.com (H.L.); 2 School of Agronomy, Honghe University, Mengzhi 661100, China; kq_biology2@126.com

**Keywords:** *Bactrocera dorsalis*, sexually immature males, methyl eugenol, sugar–vinegar–alcohol lure, attraction

## Abstract

The male annihilation technique for *Bactrocera dorsalis* relies primarily on methyl eugenol (ME), which exclusively targets mature males and consequently misses the critical window for managing sexually immature males (SIM). To address this operational gap, we cross-validated male testicular development with ME responsiveness across a 1–12-day age gradient, improved a conventional sugar–vinegar–alcohol lure (SL), and evaluated the combinations of the optimized sugar–vinegar–alcohol lure (OSL) and ME using a 2 × 2 factorial field design. Laboratory assays revealed that ME capture rates and off-white-to-yellow testicular transitions were highly synchronized and strongly correlated; notably, males aged 1–4 days exhibited entirely off-white testes and negligible ME responsiveness, establishing testicular coloration as a robust physiological hallmark of immaturity. In field trials, OSL demonstrated stable attractancy across diurnal intervals, outperforming water controls but yielding captures statistically comparable to the conventional SL. Furthermore, incorporating ME into OSL significantly enhanced SIM captures; however, factorial analysis confirmed that their interaction was not statistically significant. Ultimately, these findings indicate that OSL and ME exert a significant, independent additive effect rather than a synergistic interaction on SIM attraction. This study provides a reliable physiological–behavioral diagnostic framework and a practical, low-cost direct-mixing strategy to extend the operational window of *B. dorsalis* management.

## Simple Summary

The oriental fruit fly is a destructive pest that damages fruits and vegetables globally. Current control methods largely rely on a chemical lure called methyl eugenol, which only attracts sexually mature males. That leaves a dangerous management gap because young, newly emerged flies can freely escape traps before they mature. To solve this, we tracked the flies’ internal development. We found that the color change in male testes closely reflects their age and readiness to respond to the standard lure. We then tested a simple, low-cost bait made from sugar, vinegar, and alcohol, which naturally attracts flies of all ages looking for food. By directly mixing this food bait with the standard chemical lure, we successfully captured significantly more young, immature fruit flies in field trials. Our statistical analysis showed that these two ingredients work independently but effectively alongside each other to boost overall catches. This research provides farmers with a practical, budget-friendly trapping strategy to catch fruit flies much earlier in their life cycle, offering better crop protection and reducing economic losses for society.

## 1. Introduction

The Oriental fruit fly, *Bactrocera dorsalis*, is one of the most destructive horticultural pests worldwide, threatening the global fruit and vegetable industry and frequently causing severe economic losses in production areas due to its high fecundity, strong dispersal capability, and broad polyphagy [1,2,3]. In current integrated pest management systems, the attractant-based male annihilation technique is widely deployed [4]. This technique primarily utilizes the pheromone precursor methyl eugenol (ME) to trap adult males, thereby suppressing subsequent generations by disrupting operational sex ratios and ultimately reducing baseline population levels [5,6].

However, conventional ME-trapping techniques have a limited operational window. Because ME primarily targets sexually mature males, heavy male captures often indicate that the field population has already dispersed, colonized the area, or even partially mated [7,8,9]. From the perspective of pest population dynamics, advancing the management or monitoring window to encompass the sexually immature stage post-emergence would provide a more comprehensive understanding of field population dynamics. Therefore, investigating the behavioral characteristics, physiological traits, and attractant responses of sexually immature males (SIM) is critical for refining early-stage population management strategies for this pest.

In natural habitats, *B. dorsalis* typically exhibits a pronounced sexually immature phase, during which individuals do not display courtship or mating behaviors [10,11]. Accurately determining the sexual maturity status of individuals is a prerequisite for conducting targeted scientific research. Previous studies have mostly inferred developmental stages under laboratory conditions based on the chemotactic orientation of males to ME or their mating frequency; however, due to natural variations in individual developmental rates, purely behavioral indices can introduce ambiguity in practical applications [7,12]. Therefore, identifying a stable, visual physiological index that correlates reliably with behavioral responses is of significant practical value for precisely defining the sexually immature state of newly emerged males.

Concurrently, the daily behaviors of immature males are predominantly driven by basic survival needs, such as nutrient acquisition [13]. Although conventional SL (typically prepared from fermented beverages or condiments) are moderately attractive to both male and female adult fruit flies, optimization of blend ratios specifically tailored to SIM remains limited [14,15,16,17]. Furthermore, systematic data remain scarce regarding the field diurnal activity rhythms of this specific subpopulation, as well as the potential interactions that may occur when food-derived cues are combined with ME. Resolving these specific issues is crucial for developing early-population monitoring and mass-trapping technologies targeting SIM.

To address these gaps, this study systematically investigated these questions through a combination of laboratory and field trials. First, by cross-validating testicular developmental characteristics with ME-directed behavioral responses, we established the validity of “off-white testes” as a robust physiological indicator for identifying SIM. Subsequently, the formulation ratios of an OSL were systematically refined in the laboratory, followed by field assessments of its trapping efficacy and the diurnal activity rhythms of SIM. Finally, using a 2 × 2 factorial design, we demonstrated that OSL and ME exert a significant, independent additive effect rather than a synergistic interaction on SIM. Ultimately, these findings offer a simple, cost-effective, and pragmatic technical framework for blending attractants and achieving precise, early-stage management of *B. dorsalis*.

## 2. Materials and Methods

### 2.1. Insects

Mango fruits infested with *B. dorsalis* were collected from an abandoned orchard in Yuanjiang County, Yuxi City, Yunnan Province, China (101°58′10″ E, 23°36′03″ N) and transported to the laboratory for colonization [18]. Briefly, the laboratory rearing conditions were maintained at 25 ± 1 °C, 60 ± 10% relative humidity, and a 14:10 (L:D) h photoperiod. The infested mangoes were placed in mesh-screened containers layered with fine sand at the bottom and kept for approximately 4 weeks, allowing mature larvae to emerge from the fruit, drop into the sand, and pupate. Upon emergence, adults were transferred to acrylic rearing cages (40 cm × 40 cm × 40 cm; rearing density of ~500 flies per cage) and provided with artificial nutrition (10% yeast extract, 30% white sugar, and 1% agar) and sterile water. Once mating behavior was observed within the cages, mated females were provided with plastic cups as oviposition substrates. Eggs were collected daily and transferred into 1 L culture vessels containing a larval diet (10% yeast extract, 30% white sugar, 15% wheat bran, 1% agar, and 44% water). These vessels were placed over fine sand to facilitate larval pupation. Pupae were collected periodically and returned to adult rearing cages. Emerged adults were either co-reared at a 1:1 female-to-male sex ratio or separated by sex into distinct cages. Wild *B. dorsalis* populations were periodically introduced from the same abandoned orchard and crossed with the laboratory colony to maintain genetic diversity. Mating behavior was observed to initiate at 6 days post-emergence, and the vast majority of females had mated by 9 days of age.

### 2.2. Preparation of Attractants

The SL is a conventional lure widely used for managing fruit fly pests in China [17,19]. To prepare a 100 mL SL mixture, brown sugar (Hongmian, Guangzhou, China), aromatic vinegar (total acidity as acetic acid ≥ 5.00 g/100 mL; Hengshun, Zhenjiang, China), and Chinese Baijiu (≥53% alcohol by volume; Hongxing, Beijing, China) were added to sterile water according to the ratios specified in Table 1.

To prepare the ME-OSL solutions, various doses of ME (≥98% purity; TCI, Tokyo, Japan) were first dissolved in the Baijiu (alcohol). Brown sugar, aromatic vinegar, and sterile water were then incorporated based on the optimized formulations derived from our laboratory results, yielding ME-OSL solutions with a gradient of ME concentrations (0.1, 0.2, 0.4, 0.8, and 1.6 mg/mL). All attractants were freshly prepared approximately 1.5 h before the onset of each experiment.

### 2.3. Trap Design and Construction

**Figure 1:**
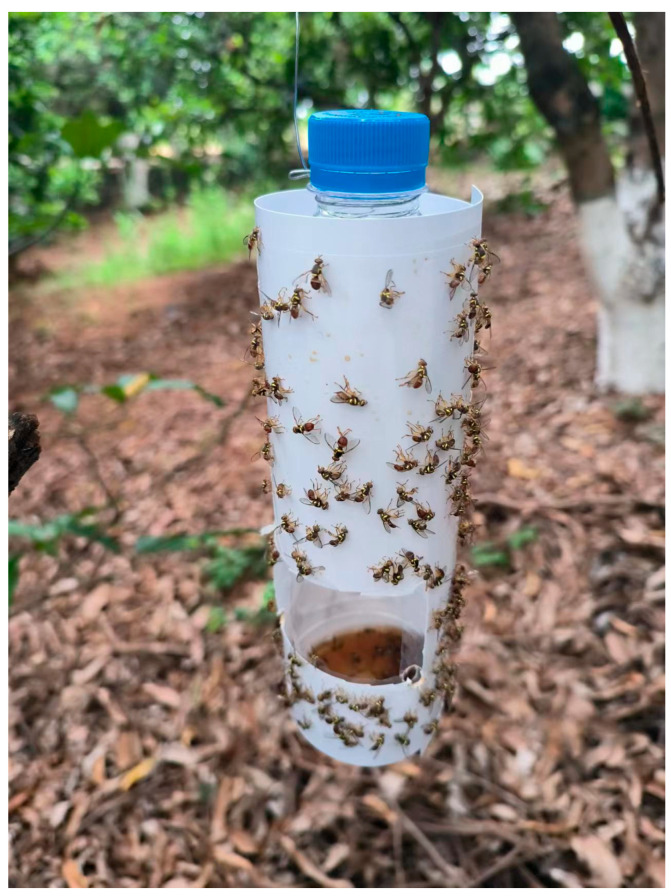
Schematic diagram and structural specifications of the 500 mL sticky-bottle trap in the field.

The traps (Figure 1) were constructed using a 500 mL plastic bottle (~20 cm in height, ~6.5 cm in diameter) wrapped externally and secured with a white sticky board (hereafter referred to as the 500 mL trap). Two opposing openings (~4 cm in length, ~2.5 cm in width) were cut through both the sticky board and the bottle wall; one opening was positioned ~4 cm from the bottle base, and the other was ~7 cm from the base. The attractant source was placed at the bottom of the bottle. Target flies responding to the chemical cues and flying toward the trap were recorded as captured if they were caught by the outer sticky board, trapped inside the bottle cavity, or drowned in the liquid lure at the bottom. Similarly, a 300 mL trap variant was constructed from a 300 mL plastic bottle (~13.5 cm in height, ~6 cm in diameter) and a white sticky board, with opposing openings located at ~2.5 cm and ~5.5 cm from the base.

### 2.4. Laboratory Assay Procedures

Virgin males kept isolated from females (1–12 days old) were selected as experimental subjects. Groups of 50 males of the same age were transferred into clean rearing cages. Following a 4 h water and nutrient deprivation period, a 250 μL aliquot of ME solution (200 mg/mL in ≥99% dichloromethane) was applied to a filter paper disc. Once the dichloromethane had completely evaporated, the filter paper was placed inside a 300 mL trap. Bioassays commenced at 10 a.m. by placing the trap in the center of the rearing cage; the trap was removed after 30 min, and the number of captured males was recorded. Four replicates were performed for each age group. Additionally, another subset of males (1–12 days old) was fixed in phosphate-buffered saline (Solarbio, Beijing, China) to dissect the testes, which were then imaged at 80× magnification under a stereomicroscope (Nikon, SMZ445, Tokyo, Japan). Fifty males were examined per age group. All aforementioned assays were conducted using the same cohort of reared insects.

To optimize SL composition, 3-day-old virgin males reared in isolation from females were used. Fifty males were transferred to a clean cage and subjected to a 4 h water and nutrient deprivation treatment. Two 300 mL traps were then placed symmetrically inside the cage (~20 cm apart); one trap contained 10 mL of the treatment lure, while the other contained 10 mL of the control lure (Table 1). The assay ran from 10 a.m. to 11 a.m. Each treatment combination was replicated six times. Prior to each replicate, the spatial positions of the treatment and control traps were swapped to eliminate potential bias from ambient light or other confounding environmental factors on insect choice behavior. At the end of each test, the traps were removed to count the captured males, thereby determining the OSL with the highest attractancy.

### 2.5. Field Trial Procedures

Field trials were conducted in the abandoned mango orchard in Yuanjiang County, Yuxi City, Yunnan Province (101°58′10″ E, 23°36′03″ N). The orchard features flat terrain planted with mango (*Mangifera indica* ‘Tainong No. 1’) at a tree spacing of 2–5 m and a tree height of ~3 m. Trials were carried out on clear days in June under the following environmental conditions: temperature 28–35 °C, relative humidity 72–85%, and mean daily wind speed < 1 m/s. Notably, these macro-climatic parameters remained largely consistent between the June 2025 and June 2026 trial windows. Shaded, well-ventilated branches avoiding direct sunlight were randomly selected as trap-hanging sites (1.5–2.0 m above the ground), with a minimum distance of ~20 m between adjacent traps. To eliminate potential position effects and spatial biases, trap-hanging sites were randomly rotated between trial dates. The field study comprised three separate trials. All trials utilized 500 mL traps filled with 10 mL of the corresponding lure. After each collection period, all traps were retrieved simultaneously to count the catches.

(1) Diurnal variation in captures: Trials were conducted on 3 June and 5 June 2025, during two test intervals (10 a.m.–12 p.m. and 4–6 p.m.) to evaluate the effect of time of day on the capture of SIM. Three treatments were evaluated: the OSL group, the SL group, and a water control group, with four replicates per treatment.

(2) Dose–response screening for optimal ME-OSL concentration: Trials were conducted from 10 a.m. to 12 p.m. on 11 June, 14 June 2025, and 15 June 2026. Six treatments were established, comprising five ME-OSL formulations with distinct ME concentrations (0.1, 0.2, 0.4, 0.8, and 1.6 mg/mL) and one OSL control group (0 mg/mL ME), with two replicates per treatment.

(3) Interaction and potential synergy between ME and OSL: Trials were performed from 10 a.m. to 12 p.m. on 17 June, 20 June 2025, and 22 June 2026. A 2 × 2 factorial design was implemented with four treatments: ME-OSL (0.2 mg/mL ME), OSL alone, ME-water (0.2 mg/mL ME), and a water control, using two replicates per treatment.

Across all field trials, only SIM were recorded; females and mature males were excluded from the analysis. The sexual maturity status of captured males was verified via laboratory dissection: individuals with distinctly yellow testes were classified as sexually mature, whereas those with off-white testes were classified as sexually immature.

### 2.6. Statistical Analysis

For laboratory assays, generalized linear models (GLMs) with a binomial distribution and logit link function were constructed using the “number of captured vs. uncaptured males” and “number of yellow vs. off-white testes males” as bivariate response variables. This study analyzed the main effect of age on capture rates and the proportion of males with yellow testes. Spearman’s rank correlation analysis was applied to evaluate the relationship between these two parameters across different ages. To optimize the lure formulation, the impact of sugar, vinegar, and alcohol components was assessed at two levels: first, GLMs were used to analyze the significance of each ingredient and estimate marginal preference probabilities; second, a selection index (SI, calculated as the difference between treatment and control captures divided by total captures) was determined for the nine combinations, with significant preferences identified using a two-tailed binomial test followed by false discovery rate (FDR) correction [20,21]. Significance for the GLMs (Type II Likelihood Ratio χ^2^ test) and post hoc pairwise comparisons (with Šidák correction) were performed using the ‘car’ and ‘emmeans’ packages in R, respectively [22,23].

For field data, generalized linear mixed models (GLMMs; ‘lme4’ package, Poisson distribution, log link function) incorporating “trial date” and “trap location” as random effects were first deployed to analyze the effects of treatment (OSL, SL, and water control), testing period (10 a.m.–12 p.m. and 4–6 p.m.), and their interaction on SIM captures (Type III Likelihood Ratio χ^2^ test) [24]. These random factors were specified to capture daily, interannual, and spatial environmental variations. Although their variance components were negligible due to homogeneous spatio-temporal backgrounds, retaining these factors ensures conservative and accurate standard error (SE) estimations for fixed effects despite limited factor levels. The same GLMM framework was subsequently applied to evaluate the fixed effect of ME concentration (Type II). Finally, a GLMM based on a 2 × 2 factorial design was constructed, defining OSL (present vs. absent/water), ME (added vs. not added), and their interaction as fixed effects to evaluate potential synergistic or additive effects (Type III). Prior to analysis, all GLMMs were verified to comply with model assumptions, successfully passing zero-inflation and overdispersion tests. Fixed-effects testing for the GLMMs and post hoc pairwise comparisons were carried out using the ‘car’ and ‘emmeans’ packages, respectively, with *p*-values uniformly adjusted using the Šidák method. All statistical workflows and figures were generated using RStudio software (R version 4.4.1).

## 3. Results

### 3.1. Laboratory Assay Results

**Figure 2:**
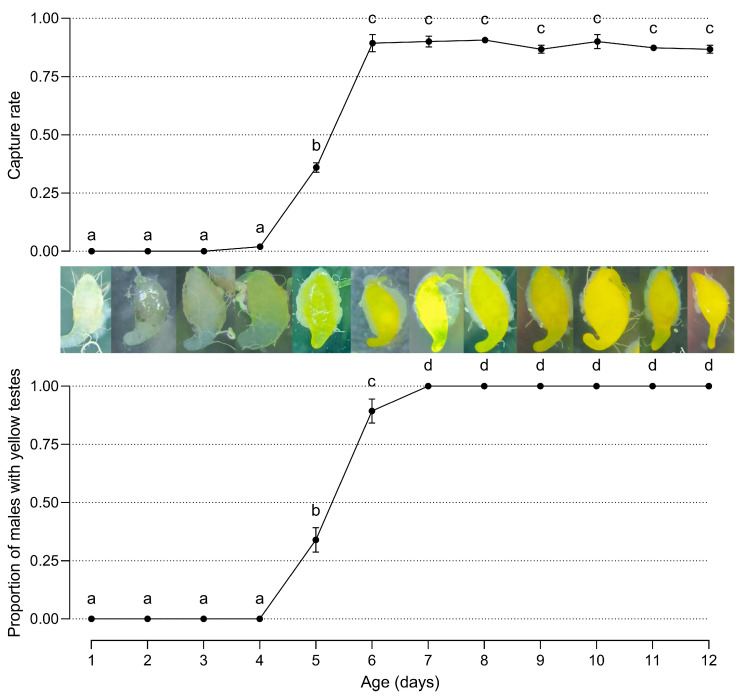
Age-dependent changes in capture rate and testis coloration of male *B. dorsalis*. The top panel displays the mean (±SE) capture rate in response to ME, while the bottom panel shows the proportion (mean ± SE) of males with yellow testes from day 1 to day 12. Representative micrographs of dissected testes for each corresponding age are presented in the middle panel. Different lowercase letters indicate statistically significant differences among ages (*p* < 0.05, Šidák’s test).

The results demonstrated that age exerted a highly significant effect on the capture rate of males attracted to ME (χ^2^ = 1513.1, df = 11, *p* < 0.001) (Figure 2). As age increased, the capture rate transitioned through three distinct developmental phases: males aged 1–4 days exhibited almost no response to ME, with a capture rate of zero or near-zero (only 0.020 ± 0.011 at 4 days of age); at 5 days of age, the capture rate rose significantly to 0.360 ± 0.039 (*p* < 0.05), though it remained significantly lower than those of subsequent age groups (*p* < 0.05); and from 6 days of age onward, the capture rate peaked and stabilized between 0.867 and 0.907.

Similarly, age significantly influenced the proportion of males possessing yellow testes (χ^2^ = 2124.6, df = 11, *p* < 0.001) (Figure 2). Specifically, the testes of males at 1–4 days of age were completely translucent and off-white. In contrast, all males successfully captured by ME (5–12 days) consistently exhibited opaque, bright yellow testes. This physiological change is macroscopically obvious and highly definitive. At 5 days of age, the testes of some individuals began turning yellow, with the proportion rising to 0.340 ± 0.039, which was significantly higher than that of 1–4-day-olds but significantly lower than subsequent age groups (*p* < 0.05). By day 6, this proportion rose sharply to 0.893 ± 0.025 (*p* < 0.05), yet remained significantly lower than that of older cohorts (*p* < 0.05). From 7 days of age onward, the testes of all examined males had completely turned yellow. Notably, no developmental abnormalities, such as testicular deformities, concealed/unidentifiable coloration, or unilateral yellowing, were observed throughout the evaluation. Correlation analysis further revealed a highly significant and strong positive correlation between male capture rates and the proportion of individuals with yellow testes (r = 0.847, *p* < 0.001), indicating that their behavioral responses and physiological traits are highly synchronized as age increases.

**Figure 3:**
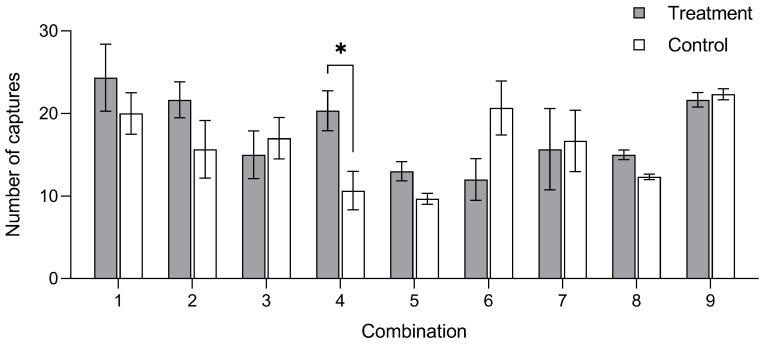
Behavioral responses of SIM *B. dorsalis* (3 days old) to different SL formulations. Data present the number of captures (mean ± SE) in traps baited with the nine tested combinations (Treatment, grey bars) versus the conventional SL (Control, white bars). The asterisk (*) indicates a statistically significant difference between the treatment and control within the same combination (P_FDR_ < 0.05).

Among the three primary ingredients of SL, vinegar had the most pronounced effect on the field capture of SIM (χ^2^ = 10.48, df = 2, *p* < 0.01), followed by sugar (χ^2^ = 8.16, df = 2, *p* < 0.05) (Figure 3). In contrast, alcohol did not significantly alter capture rates (χ^2^ = 1.10, df = 2, *p* > 0.05). Estimated marginal means indicated that the highest predicted selection probabilities for males toward the treatment group—0.575, 0.603, and 0.550—were achieved when sugar, vinegar, and alcohol were set to 4, 12, and 18, respectively. Among all tested formulations, only the “sugar 4 + vinegar 12 + alcohol 15” combination exhibited significant positive attractant activity toward the males (SI = 0.312, P_FDR_ < 0.05); no significant differences were observed between the treatment and control groups for the remaining eight blends. Consequently, the “sugar 4 + vinegar 12 + alcohol 15” formulation was selected as the optimal composition for OSL in this study.

### 3.2. Field Trial Results

**Figure 4:**
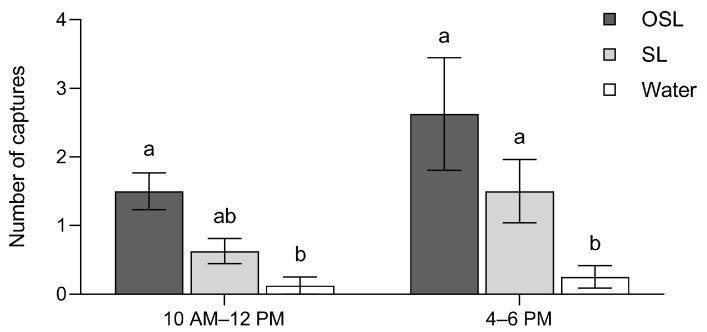
Number of SIM *B. dorsalis* captured by traps with different treatment solutions at different daily intervals. Values are expressed as mean ± SE. Different lowercase letters denote statistically significant differences among treatments (OSL, SL, and Water) within the same time period (*p* < 0.05, Šidák’s test).

Lure treatment significantly affected the captures of SIM in the field (χ^2^ = 11.23, df = 2, *p* < 0.01). In contrast, neither the testing period (χ^2^ = 0.34, df = 1, *p* > 0.05) nor the lure × testing period interaction (χ^2^ = 0.25, df = 2, *p* > 0.05) reached statistical significance (Figure 4). During the morning interval (10 a.m.–12 p.m.), OSL yielded the highest male captures (1.50 ± 0.43), which was significantly greater than the water control (*p* < 0.05) but statistically comparable to SL. Similarly, in the afternoon (4–6 p.m.), OSL captured the most males (2.63 ± 0.57); this value did not differ significantly from SL, but both lures outperformed the water control (*p* < 0.05). In summary, OSL demonstrated robust and stable attractant activity in the field during both testing periods. However, its field efficacy did not significantly surpass that of SL as it did in the laboratory bioassays.

**Figure 5:**
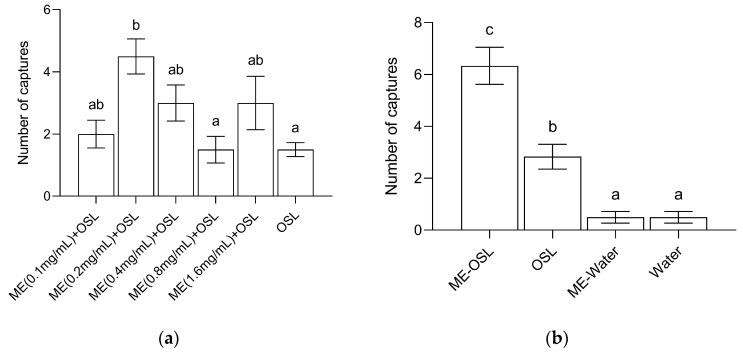
Effects of ME combination with OSL on the capture of SIM *B. dorsalis*. (**a**) Mean (± SE) number of males captured by traps baited with ME-OSL mixtures at varying ME concentrations (0 to 1.6 mg/mL). (**b**) Field factorial bioassay evaluating the independent and joint effects of OSL (present vs. water) and ME (added vs. not added). Different lowercase letters indicate statistically significant differences among treatments within each panel (*p* < 0.05, Šidák’s test).

The incorporation of varying concentrations of ME into OSL significantly altered the captures of SIM (χ^2^ = 14.62, df = 5, *p* < 0.05) (Figure 5a). The highest male capture rate was recorded at an ME concentration of 0.2 mg/mL (4.50 ± 0.87), which was significantly higher than the OSL baseline (0 mg/mL ME) and the 0.8 mg/mL ME-OSL treatment (*p* < 0.05), but did not differ significantly from the other concentration groups. Thus, 0.2 mg/mL was established as the optimal concentration of ME for the ME-OSL blend.

Both the presence or absence of OSL (χ^2^ = 7.67, df = 1, *p* < 0.01) and ME (χ^2^ = 7.60, df = 1, *p* < 0.01) exerted highly significant main effects on the field captures of SIM (Figure 5b). However, their interaction was not statistically significant (χ^2^ = 0.86, df = 1, *p* = 0.354), demonstrating that OSL and ME operate via independent additive effects to enhance trap captures. The combined ME-OSL treatment achieved the highest captures (6.33 ± 1.03), outperforming OSL alone (2.83 ± 0.69, *p* < 0.05), while both treatments captured significantly more males than ME alone or the water control (both 0.50 ± 0.29, *p* < 0.05).

## 4. Discussion

Wild *B*. *dorsalis* populations are generally understood to undergo a sexually immature period of 15–20 days, during which individuals refrain from mating and oviposition [25,26,27,28]. Because current conventional management technologies rely heavily on ME, which exclusively targets sexually mature males, the operational window for early-stage population control is frequently missed. Consequently, accurately characterizing the sexually immature status of males and developing targeted lure strategies could provide a valuable, proactive supplement to the early population management of this pest.

This study demonstrates that male sexual maturity can be accurately diagnosed via the visual examination of testicular coloration, offering a simple and highly efficient physiological method for field population monitoring and behavioral research on *B. dorsalis*. Our results showed that the male capture rate by ME exhibited a trend of an initial significant increase followed by a plateau as age progressed (Figure 2). This developmental timeline differs slightly from the findings of Shelly et al. (who reported stabilization at 15 days of age), a discrepancy that may stem from variations in laboratory rearing conditions (e.g., nutrient availability, colony density) or the distinct genetic backgrounds of the tested insect populations, although the overall age-related trajectory remains fundamentally consistent [7]. The capture rate of males aged 6 days or older stabilized between 86.7% and 90.7%, falling short of the theoretical 100%. This ceiling effect might be attributed to the confined space of laboratory bioassay cages, where the background concentration of ME became saturated during the later stages of the experiment, thereby masking the volatile odor plume. This saturation likely disrupted the orientation and localization behaviors of some males, ultimately preventing their capture [29,30,31]. Similarly, the proportion of males possessing yellow testes followed an age-dependent trajectory of an initial increase followed by a plateau, maintaining a 100% saturation from day 7 onward. Correlation analysis confirmed that the age-related trends of male ME capture rates and the proportion of yellow testes were highly synchronized, with both parameters remaining at zero during days 1–3. Therefore, when diagnosing the sexually immature status of *B. dorsalis* males, anatomical examination of testicular color (a physiological marker) can reliably substitute or supplement ME chemotaxis (a behavioral index). Notably, while the testes of all 4-day-old males were off-white, a minute fraction of individuals exhibited a weak response to ME (capture rate: 0.020 ± 0.011). Statistical analysis revealed that the capture rate at day 4 did not differ significantly from that at days 1–3 (0%), suggesting that these captures were not driven by active chemotaxis but rather represented incidental interceptions during random flight and landing events [32].

Furthermore, while previous studies utilizing ME chemotaxis or mating propensity could ensure that the vast majority of males beyond a certain age threshold were sexually mature, the occurrence of ME responses in low-age individuals is more likely attributable to individual variations in development rates (leading to precocious maturity) rather than an intrinsic chemotactic response in truly immature insects [7,26]. In contrast, males aged 1–4 days in the present study exhibited distinct age-specific fidelity, showing no significant behavioral response to ME alongside exclusively off-white testes. This cross-validation of physiological and behavioral metrics explicitly establishes “off-white testes” as the core physiological hallmark defining sexually immature individuals that lack ME responsiveness in *B. dorsalis*.

Additionally, this study systematically optimized the component ratios of SL. Our findings indicate that fine-tuning the concentrations of brown sugar and vinegar significantly alters the attractancy of the lure toward SIM, confirming that males at this developmental stage possess high sensitivity to the volatile profiles of the sugar and vinegar components (Figure 3). However, in field trials, although OSL captured more SIM than SL, the difference did not reach statistical significance. This discrepancy likely arises from the substantial “complex olfactory background” inherent in complex field habitats (such as blended volatiles from host mangoes and surrounding non-host vegetation). These complex ambient odors may have masked or interfered with the ability of immature males to discriminate the subtle differences in the chemical profiles of the two lures, thereby diluting the competitive advantage of the formulation optimized under laboratory conditions [33,34,35].

Furthermore, different field testing intervals (10 a.m.–12 p.m. vs. 4–6 p.m.) exerted no significant effect on the capture of SIM, implying that the diel activity patterns of immature individuals diverge significantly from those of their mature counterparts (Figure 4). The diurnal activity rhythms of sexually mature males are well known to be regulated primarily by ambient light intensity and driven by mate-seeking and courtship behaviors [36]. In contrast, the behavior of SIM is likely governed by basic survival imperatives, such as nutrient acquisition and predator avoidance, resulting in a more uniform behavioral distribution throughout the day [37].

Field trials further demonstrated that the incorporation of ME into OSL significantly enhanced its attractancy toward SIM (Figure 5b). Although the 2 × 2 factorial analysis revealed that their interaction was not statistically significant—meaning it did not exhibit synergy as strictly defined in chemical ecology—the two components exerted a significant, independent additive effect on increasing trap catches. The biological mechanism underlying this independent additive effect may be closely linked to the functional segregation of their chemosensory pathways. Although ME functions as a pheromone precursor and serves as a direct precursor to the primary component of the sex pheromone released by mature males during dusk wing-fanning courtship, it is also widely synthesized in the floral scents of numerous plants, acting as an environmental orientation and localization cue for insects seeking plant-derived nutrients, such as nectar or damaged fruits [38,39,40]. Consequently, from a molecular neurobiological perspective, we propose as a working hypothesis that the chemotactic responses of SIM elicited by OSL (conventional food-derived cues) and ME (plant-derived kairomonal signals) might be mediated by distinct peripheral olfactory receptor pathways [41,42,43], a proposition that warrants future electrophysiological and neurobiological validation.

This independent additive effect provides preliminary evidence that OSL and ME possess complementary management potential in the field, offering a simple, low-cost, and direct-mixing framework for the conventional control of *B. dorsalis*; however, the long-term stability and efficacy of its large-scale application warrant further validation. Additionally, the absolute numbers of SIM captured across all treatments in our field trials were relatively low. This low capture was likely because the trials coincided with the fruiting period of the orchard, a seasonal window when sexually mature individuals heavily dominate the field population structure, thereby compressing the relative proportion of the immature subpopulation [44,45].

## 5. Conclusions

This study establishes “off-white testes” as a reliable physiological indicator for identifying SIM of *B. dorsalis*, cross-validating internal physiological traits with behavioral responses. Field trials demonstrated that while the trapping efficacy of the OSL was statistically comparable to the conventional SL, its direct combination with ME exerted a significant, independent additive effect on trap catches. However, a major constraint of this study is the low absolute number of SIM captured across all field treatments, which reflects a clear limitation in immediate trapping volume. Therefore, rather than serving as a definitive or optimized management tool at this stage, this low-cost direct-mixing strategy offers a preliminary framework whose long-term stability and operational efficacy in large-scale field applications warrant further rigorous validation.

## Figures and Tables

**Table 1:** Formulations and ingredient concentrations of the ten SL.

Combination	Components of Lure			Final Volume of Lure (mL)
	Brown Sugar (g)	Aromatic Vinegar (mL)	Chinese Baijiu (mL)	
1	6	12	12	100
2	4	10	18	100
3	6	8	18	100
4	4	12	15	100
5	5	12	18	100
6	6	10	15	100
7	5	10	12	100
8	5	8	15	100
9	4	8	12	100
CK ^1^	6	10	20	100

^1^ CK represents the control group, which is the conventional formulation used for *B. dorsalis* management.

## Data Availability

The original data are included in the paper. Further inquiries can be directed to the corresponding authors.
